# Cases of Puerperal Convulsions

**Published:** 1851-05

**Authors:** Joel E. Hendrick

**Affiliations:** Auburn, Indiana


ARTICLE IV.

*Cases of Puerperal Convulsions.* By JOEL E. HENDRICK, M. D., of Auburn, Indiana.

CASE 1. I was called on the morning of the 14th of August 1846, to see Mrs. Hash, aged about 25 years, who was supposed to be in labor with her first child. On my arrival at 9 o'clock A. M., I found her laboring under pretty severe pains, which occurred regularly at intervals of about five minutes. I therefore immediately made an examination per vagina, and found the os uteri rigid and not sufficiently dilated to admit the point of a finger. I learned on inquiry, that the bowels had been well evacuated. I therefore, for the purpose of facilitating the dilation of the os uteri, drew from the arm about 16 oz. of blood, and afterward administered a full dose of morphine, in consequence of which the pains were diminished in force and frequency, recurring in a moderate degree, at intervals of about half an hour, until about 3 o'clock P. M., when they again commenced to increase. On examination, the os uteri was now found to be less rigid than formerly, and sufficiently dilated to admit the passage of a finger. The pains gradually increased from this time, and the os uteri slowly dilated, until about 8 o'clock P. M., when she was suddenly seized with a very severe convulsion. She was immediately bled to the extent of about 24 oz., but as the convulsions continued to occur, the os uteri being now sufficiently dilated to admit the passage of the hand, and the head of the child having not yet entered the superior strait, I concluded to deliver immediately by turning. I therefore, after placing the patient in a proper position, introduced my hand, seized and brought down one foot, and delivered without much delay a living male child. The placenta was soon after detached and expelled, and the convulsions immediately subsided. Both the mother and child lived and did well.

Case 2. I was called on the evening of the 2d of March, 1851, to see Mrs. Keen, a large plethoric woman aged 27 years. I arrived at 12 o'clock P. M., and found on inquiry, that the patient was supposed to be at about the commencement of the ninth month of her first pregnancy; that for the last 48 hours she had suffered from very intense pain in the head; and that at about 6 o'clock P. M., of the same day, she was seized with a very severe convulsion, which had occurred at intervals of from half an hour to an hour, leaving her during the intervals, entirely insensible.

Her breathing was now stentorous, her head hot, extremities cold, and pulse frequent and feeble. The tongue had been badly lacerated during the convulsions, and was dreadfully swollen. I immediately opened a vein in the arm, but succeeded in obtaining but a very few ounces of blood.

As the convulsions continued to occur, and as I was informed by the women present, that there had been a discharge several hours previous to my arrival supposed to be the liquor amni, and also that no motion of the child had been felt for several days, in consequence of which the patient herself had supposed the child to be dead, I therefore determined, if practicable, to deliver immediately. On examination per vagina, I found the os uteri soft and dilated to about the size of a dollar, and the head of the child presented at the superior strait. I concluded therefore to deliver by turning without further delay. After placing the patient in a proper position, I slowly and cautiously introduced my hand, seized and brought down one foot, and delivered with a moderate amount of traction, a dead male child. The placenta was separated by the contractions of the uterus, and immediately extracted from the vagina. The delivery was completed at one o'clock on the morning of the 3d, about an hour after my arrival.

The patient remained insensible during the operation of turning, and until her death, which took place at 4 o'clock, three hours after delivery was effected, the convulsions having continued to occur at about the same intervals as before her delivery.

Of the othei two cases of puerperal convulsions that have come under my observation, no notes were taken at the time. I will therefore merely state, that in one case the convulsions made their appearance during labor, and after the child had passed the superior strait. The case occurred in the summer of 1844, and the patient, Mrs. Gilbert, was about 35 years of age, and in labor with her fourth child. On the incursion of convulsions, she was bled largely, and although the convulsions continued to occur, she was speedily delivered, without manual assistance, of a living female child. Both the mother and child lived and did well.

The other case occurred in the practice of Dr. K——, in the winter of 1849. I was called to see the patient in consultation with Dr. K., and learned, on inquiry, that she was about 22 years of age, and supposed to be at about the commencement of the seventh month of her first pregnancy; that she had been suddenly seized with convulsions without any premonitory symptoms about twelve hours previous to my arrival; that the convulsions at first occurred about every fifteen minutes, but that after a full bleeding they had diminished in frequency to about one per hour. She was now, and had been from the first attack, entirely insensible.

As the friends were very anxious that she should be immediately delivered, I made an examination per vagina, but found that dilatation of the os uteri had not commenced. We therefore directed that a brisk cathartic should be administered, and that after the bowels had been thoroughly evacuated, opiates should be given.

I did not see the patient afterward, but was informed by Dr. K., that after the operation of the cathartic, the convulsions subsided, and that during the next day, labor came on regularly, and she was delivered, without manual assistance, of a dead child. The woman soon recovered.

